# Facilitator experiences and lessons learned from the Betel nut intervention trial (BENIT)

**DOI:** 10.1186/s12889-024-17788-4

**Published:** 2024-01-24

**Authors:** Gena A. Rojas, Silvera Erari, Yvette C. Paulino, Thaddeus A. Herzog

**Affiliations:** 1 University of Guam Cancer Research Center, University of Guam, University Dr, House 7, Dean’s Circle UOG Station, 96923 Mangilao, GU USA; 2 University of Hawaii Cancer Center, 701 Ilalo St, 96813 Honolulu, HI USA

**Keywords:** Betel quid, Cessation intervention, Facilitator experiences, Protocol adaptation

## Abstract

**Background:**

The Betel Nut Intervention Trial (BENIT; ClinicalTrials.gov - NCT02942745) is the first known randomized intervention trial specifically designed for areca nut chewers in the western Pacific region who want to quit. The current study is a separate, exploratory study that examined the experiences of the BENIT facilitators during its implementation in Guam and Saipan of the Northern Mariana Islands and the extent to which the BENIT protocol was adapted to meet the participants’ and facilitators’ needs.

**Methods:**

All six BENIT program facilitators completed an online survey consisting of quantitative (Likert scale) and qualitative (open-ended) questions. Survey items were grouped as follows: facilitator demographics, prior training and preparation, BENIT experience, beliefs about the program’s effectiveness, and beliefs about protocol adaptations.

**Results:**

Most of the facilitators felt prepared to deliver the BENIT program after several weeks of moderately intensive training. Facilitators felt the BENIT program was generally effective and that the “trigger logs” and “self-monitoring logs” worked as intended. However, they also noted that more time and support would have been helpful to overcome some of the obstacles inherent to implementing a novel program.

**Conclusion:**

The current findings can be used to inform, modify, and tailor subsequent areca nut cessation programs in Micronesian communities and to improve future versions of BENIT.

## Background

Areca nut (AN), the fruit of the *Areca catechu* palm, and betel quid (BQ), the AN wrapped in a betel leaf with a variety of ingredients including smokeless tobacco and slaked lime, are carcinogenic substances associated with cancer of the head and neck region [1]. AN and BQ (ANBQ) are chewed by an estimated 600 million people worldwide [2], including those in the Western Pacific region. In Guam and Saipan, ANBQ is colloquially referred to as “betel nut.” This term is often used in the current study, particularly in reference to study materials, such as the survey. Thus, for the current study, ANBQ and “betel nut” should be considered synonymous.

Guam is an unincorporated island territory of the U.S. in Micronesia with a population of 159,348 [3] comprised of indigenous CHamorus, who account for 37.3% of the population, followed by 26.3% Filipinos, 7.1% Caucasians, and 7% Chuukese; the remaining resident percentages are made up mostly of other Asian and Pacific Islanders [3]. Saipan is part the Commonwealth of the Northern Mariana Islands (CNMI) in Micronesia, which also includes Tinian and Rota. The CNMI has a population of 47,329, with the top four ethnic groups comprised of Filipinos (32.7%), CHamorus (25.4%), Other Native Hawaiians and Pacific Islanders (7.1%), and Chinese (6.9%). In Micronesia, the ANBQ chewing prevalence ranges from 3% in the Republic of the Marshall Islands to 94% in Yap; in Guam, the prevalence has been estimated at 11%, [4] and in the CNMI, 24% [5]. There are no chewing prevalence estimates specific to Saipan.

Research on ANBQ cessation is increasing [6] but is still modest compared other research literatures, such as that for smoking cessation. This literature gap prompted the University of Guam and University of Hawaii Cancer Center Partnership to develop a ANBQ cessation program, ultimately named the Betel Nut Intervention Trial (BENIT; registered at ClinicalTrials.gov on 24/10/2016, protocol NCT02942745) [7, 8]. Preparation for developing the BENIT included identifying high-risk groups in Micronesia [9], developing BQ dependence tools [10–12], and identifying AN biomarkers [13].

The BENIT is a cognitive-behavioral change intervention program aimed at helping ANBQ chewers in Micronesia and the rest of the Western Pacific region quit chewing. The program was pilot tested in Guam in 2014 [14], then implemented fully six years later in Guam and Saipan [7, 8] using a detailed treatment manual. Given the unprecedented nature of the program and the limited number of individuals in Guam and Saipan with extensive experience leading behavior change interventions, program facilitators were encouraged to tailor the intervention to the needs of individual participants while also adhering to the general framework described in the treatment manual. We sought to learn about the challenges, successes, and lessons learned by the facilitators of this novel program.

## Methods

The aim of this study was to evaluate the experiences of the BENIT facilitators during its implementation in Guam and Saipan, and to examine the extent to which the BENIT protocol was adapted to meet the needs of the program’s participants and facilitators.

### Betel nut intervention trial (BENIT)

The BENIT is a cognitive-behavioral change intervention program consisting of five hour-long, in person sessions conducted over a 22-day period. The first two sessions were designed to prepare chewers to make a quit attempt on “quit day”, which occurred during session 3 (15 days after program commencement). Sessions 4 and 5 focused on helping chewers stay quit (i.e., prevent relapse). Full details of the BENIT design can be found elsewhere [7]. The BENIT 22-day results revealed a significant intervention effect, with 38.6% reporting cessation in the intervention group compared to 9.1% cessation in the control group [8].

BENIT facilitators were trained in the classroom for several weeks leading up to the program’s commencement. Training included several mock sessions (i.e., simulated practice sessions) with feedback from the principal investigators (PIs), a one-day course on Brief Tobacco Intervention provided by the Guam Department of Public Health and Social Services and, for Guam facilitators, a three-day training course in Nicotine Cessation Facilitation.

The BENIT was modeled after a cigarette-smoking cessation program in the U.S. mainland [15], but adapted to account for differences between (1) cigarette smoking and ANBQ chewing, and (2) Guam and Saipan cultures and the U.S. mainland cultures. For example, the BENIT’s cognitive component addressed chewers’ attitudes towards ANBQ chewing, and educated chewers on how ANBQ chewing increases their risk for oral cancer. The PIs trained facilitators who were familiar with the customs and languages of the BENIT participants. Additional cultural adaptions of the BENIT included identifying community champions to enhance recruitment and being flexible to accommodate for “island time” (i.e., delayed session start times).

Survey items were grouped as follows (and described in detail below): facilitator demographics and prior training and preparation, facilitator experience with the BENIT, program effectiveness beliefs, and protocol adaptation beliefs. Some survey items were quantitative and employed Likert scales; other survey items were qualitative and open-ended. Both types of data collection were used to identify themes that are presented in the results section. All data were recorded, stored, and secured in password-protected files on QuestionPro and Excel.

### Measures

#### Facilitator demographics and prior training and preparation

All questions were in quantitative format. Facilitators were asked about their age, ethnicity, gender, and occupation. Two items were employed to measure the facilitators’ experiences prior to the BENIT training: (a) “Prior to facilitating BENIT, what was your level of experience in facilitating group discussion?” and (b) “Prior to facilitating BENIT, what was your level of experience in behavioral interventions?” Response options were: “not experienced,” “slightly experienced,” “moderately experienced,” “very experienced,” and “completely experienced.” Two additional items assessed aspects of the BENIT facilitator training program. The first item read, “How long did you spend in the cessation training before you participated in BENIT as a facilitator?” Responses options were: “1–3 weeks before implementation,” “1 month before implementation,” “2 months before implementation,” and “3 months or more before implementation.” The second item read, “Did the facilitator training prepare you for anticipated obstacles you may have encountered in delivering the program?” Responses options were: “yes,” “no,” and “somewhat.”

### Facilitator experience with the BENIT

#### Overall program experiences

Quantitative and qualitative survey items were employed to evaluate the facilitators’ overall program experience, and included assessments of their preparedness, perceived level of support, time allocation, obstacles and highlights as facilitators, and levels of enjoyment in program delivery.

*Preparedness*. Two items assessed the facilitators’ relative preparedness to lead the BENIT sessions: (a) “Which of the following BENIT cessation sessions did you feel most prepared in delivering the program?” and (b) “Which of the following BENIT cessation sessions did you feel the least prepared in delivering the program?” Response options were: “Session 1 Day 1,” “Session 2 Day 8,” “Session 3 Day 15 Quit Day,” “Session 4 Day 18,” and “Session 5 Day 22.” Multiple response selections were permitted.

*Support*. Two items were employed to measure the facilitators’ perceived level of support: (a) “What was the level of support provided to strengthen your facilitation skills during program delivery?” and (b) “What was the level of support provided to address obstacles during program delivery?” Response options were: “no support,” “some support,” “moderate support,” “significant support,” and “complete support.”

*Time Allocation*. Two items were employed to measure the adequacy of time allocation. The first question read, “What BENIT sessions needed more time?” Responses options were: “Session 1 Day 1,” “Session 2 Day 8,” “Session 3 Day 15 Quit Day,” “Session 4 Day 18,” and “Session 5 Day 22.” Multiple response selections were permitted. The second question read, “Was there sufficient time allotted for all BENIT cessation sessions?” Response options were: “mostly yes,” “yes,” and “no.” Only a single response selection was permitted.

*Obstacles and Highlights*. Two open-ended items were employed to measure obstacles and highlights of the program: (a) “What obstacles did you encounter?” and (b) “Please add any comments about training and preparation of the program that you would like us to know.”

*Enjoyment*. One item was employed to assess the level of enjoyment in program delivery: “How much did you enjoy delivering and facilitating the program?” Response options were: “did not enjoy,” “somewhat enjoyed,” “moderately enjoyed,” “highly enjoyed,” and “completely enjoyed.”

### Program effectiveness beliefs

Program effectiveness was assessed for both the overall BENIT program and the specific BENIT sessions. In addition, the intervention components of the BENIT participants’ “trigger logs” and “self-monitoring logs” were evaluated separately. Both quantitative and qualitative survey items were employed.

Two items assessed the facilitators’ beliefs about the overall BENIT program effectiveness: (a) “How effective do you think the cessation program was overall?” and (b) “Do you feel that the program content was an effective curriculum to reduce and stop betel nut chewing?” Responses options were: “not effective,” “slightly effective,” “moderately effective,” “very effective,” and “completely effective.” Two items were employed to measure specific BENIT session effectiveness: (a) “Which session(s) do you feel was the most effective part of the curriculum?” and (b) “Which session(s) do you feel was the least effective part of the curriculum?” Response options were: “Session 1 Day 1,” “Session 2 Day 8,” “Session 3 Day 15 Quit Day,” “Session 4 Day 18,” and “Session 5 Day 22.” Multiple response selections were permitted.

For the effectiveness of trigger logs and self-monitoring logs, two quantitative questions and two qualitative questions were employed. The quantitative questions were: (a) “Do you feel the trigger logs were effective?” and (b) “Do you feel the self-monitoring logs were effective?” Responses options were: “not effective,” “slightly effective,” “moderately effective,” “very effective,” and “completely effective.” The qualitative questions were: (a) “In your experience how were the trigger logs effective?” and (b) “In your experience how were the monitoring logs effective?”

### Protocol adaptation beliefs

#### Evaluations of protocol adaptations

The extent of and perceived acceptability toward BENIT protocol adaptations were assessed using a series of quantitative survey items. The extent of changes made was assessed by the question: “How different was the program as written from the way you delivered it?” Response options were: “no difference,” “slightly different,” “moderately different,” “very different,” and “completely different.”

Three items were employed to measure the acceptability of protocol adaptations: (a) “How acceptable do you think it is to add information or materials that the written program did not address?”, (b) “How acceptable do you think it is to leave out specific material in the written program?”, and (c) “How acceptable do you think it is to adapt the program as you became more experienced in delivering the program over time?” Responses options were: “not acceptable at all,” “somewhat acceptable,” “moderately acceptable,” “very acceptable,” and “completely acceptable.”

Two items were employed to assess adaptations to individual BENIT sessions. The first item read, “Which sessions were you most likely to adapt?” Response options were: “Session 1 Day 1,” “Session 2 Day 8,” “Session 3 Day 15 Quit Day,” “Session 4 Day 18,” and “Session 5 Day 22.” Multiple response selections were permitted. The second item read, “What were the reason(s) for adaptations?” Response options were: “because you disagree with the content,” “because you feel your personal expertise or experience has something to add,” “because of the specific attribute of the clientele,” “not enough time,” and “some things you can learn only by experience - you have to try it out first to see what works best.” Multiple response selections were permitted.

Finally, facilitators were asked to reveal any adaptations they made that were not addressed by the survey items with the question: “What other adaptations did you make that were not covered in this survey?” This was an open-ended qualitative item.

## Results

### Facilitator demographics

All six facilitators completed an online survey on QuestionPro in the summer of 2021, after the BENIT ended. Three of the six facilitators identified as CHamorro, one as Chuukese, one as multi-ethnic, and one as “other” ethnicity. There were five females and one male. Four facilitators were in the age range of 28–30 years, one in the 45–50 age range, and one above the age of 60. Facilitators had a range of job titles including program leader, graduate research assistant, research associate, non-communicable disease researcher, and self-employed. Regarding the level of experience in facilitating group discussions, two facilitators were very experienced, one was moderately experienced, two were slightly experienced, and one had no experience. Regarding the level of experience in behavioral interventions, three facilitators were not experienced, while the other three ranged from slightly to very experienced.

Four facilitators indicated spending three or more months training before implementing the BENIT, whereas one facilitator spent two months training, and another, 1–3 weeks training. Five facilitators thought the training prepared them somewhat for the obstacles they may encounter during program delivery, whereas one facilitator felt fully confident the training would prepare themself for potential obstacles.

### Facilitator experience with the BENIT

#### Overall program experiences

Table 1 provides descriptions of the five intervention sessions in addition to quantitative data about facilitator preparedness for each session. All six participants felt “most prepared” for sessions 1 and 2, whereas five of six felt “most prepared” for sessions 3, 4, and 5. Zero participants felt “least prepared” for sessions 1, 2, and 5, whereas one participant was “least prepared” for session 4 and two participants were “least prepared” for session 3. In summary, feelings of preparation were relatively high according to these data, though perceived preparedness was slightly lower in the case of session 3.

**Table 1:** Facilitator preparedness for BENIT sessions

Session Descriptions	Most Prepared	Least Prepared
Session 1 (Day 1): Ground rules, informed consent; health effects; introduction and discuss trigger logs, betel nut fading.	6	0
Session 2 (Day 8): Review logs; discuss self-management, lifestyle changes, fake chew, excuses for not chewing; quit day reminder.	6	0
Session 3 (Day 15) Quit Day: Remind and discuss withdrawal symptoms, coping strategies; plan to maximize support for non-chewing; recommend physical activity	5	2
Session 4 Day 18: Continued discussion: quitting experience, coping with triggers, high risk-situations, short term benefits, negative effects, and additional strategies for with urges to chew	5	1
Session 5 Day 22: Continued discussion: overall quitting experience, relapsing and quitting again; introduce strategies managing thoughts that can lead to relapse; Review lifestyle changes; reinforce using excuses for not chewing; employing fake chew	5	0

*Note*: Values represent the number of facilitators selecting each session

Table 2 provides quantitative data regarding the level of support received by facilitators. All six facilitators expressed that at least some level of support was provided to strengthen their facilitation skills during program delivery; three facilitators indicated significant support, and the other three indicated some, moderate, or complete support. Similarly, all six facilitators indicated that at least some level of support was provided to address obstacles during program delivery; two indicated moderate support, two indicated significant support, and the other two indicated either some or complete support.

**Table 2:** Level of support provided during program delivery

Level of Support	To strengthen facilitation skills	To address obstacles
No Support	0	0
Some Support	1	1
Moderate Support	1	2
Significant Support	3	2
Complete Support	1	1

*Note*: Values represent the number of facilitators selecting each session

Regarding time allocation for administering the BENIT program, four facilitators agreed that there was sufficient time allotted, whereas two facilitators indicated that there was “mostly” sufficient time allotted. When asked if more time was needed for each specific session, three facilitators indicated that more time was needed for session 1, whereas four, one, two, and two facilitators indicated that more time was needed for sessions 2, 3, 4, and 5, respectively.

Table 3 provides information related to obstacles and highlights from the BENIT program. Several facilitators noted “burnout”, and indicated that administrating the BENIT protocol was very demanding, both emotionally and mentally. “Burnout” was described in varied ways, such as the mental and physical effects of the typhoon Yutu, which hit Saipan during the BENIT, stress from home and work, and dealing with multiple and separate groups trying to overcome addiction simultaneously. One facilitator indicated that their dual role of facilitator and research assistant caused a substantial degree of stress and that compartmentalization of responsibilities was needed to be effective. Facilitators felt they needed more experience in delivering “harm reduction strategies.” Programmatic issues such as transportation, cultural and language barriers, and environmental issues such as a typhoon were also noted. As for the highlights of the program, facilitators cited that the training was a key factor to prepare them to deliver the program. Support from program managers was an important factor when encountering administrative issues and program obstacles, and participating in mock training sessions helped them to prepare for the actual program implementation.

Despite their obstacles and challenges, facilitators generally derived satisfaction from delivering the BENIT program, with three facilitators “completely” enjoying the process and two “highly” enjoying it.

**Table 3:** Quotations regarding obstacles and highlights of BENIT program

Obstacles		Highlights
**Burnout**: I was not prepared for the mental and emotional toll. Dealing with multiple and separate groups trying to overcome addiction simultaneously. I also felt that I had to do a lot of research independently to be better prepared to lead groups. **Inadequate Skill**: The biggest obstacle I faced was feeling inadequate to deliver harm reduction strategies given that I had no experience quitting anything, let alone a specific substance even after being trained. **Cultural Barriers**: There were cultural barriers and language barriers. **Administrative**: Transportation, recruitment, and changes in contact information was a challenge. No one could have anticipated a devastating typhoon during BENIT which impacted our participants and my ability to implement the study.		**Training**: The training was actually excellent nicotine cessation training, but it differed because we dealt with betel nut chewers. I definitely had to learn about the cultural significance betel nut was and how to navigate the conversation around that, among other things. I also feel like the facilitator, even with no experience, should already be empathetic and a quick thinker in order to make the best of the script provided. **Support**: The Tobacco Cessation program was very helpful, everything that was taught in that program we have experienced during my facilitation. It really prepared and helped me during our interventions. **Piloting**: It was great to conduct a mock training prior to implementation. It gave us an idea as to how the session would be. It also gave us the opportunity to adjust. The initial training took place at UOG and I was able to continue with the training with test subjects. This was all very helpful.

### Program effectiveness

Five out of six facilitators found the BENIT to be moderately effective, while one facilitator found it to be completely effective. Four out of six facilitators found the program content to be moderately effective in reducing and stopping betel nut chewing, whereas the other two facilitators found the program content to be very effective.

All sessions were identified as “most effective” by at least by two facilitators. Five facilitators found sessions 2 and 3 to be most effective. Session 4 was considered the “least effective” by three of the facilitators.

Three facilitators found the trigger logs to be completely effective, whereas one rated the trigger logs as very effective, and two as moderately effective. Two facilitators each found the self-monitoring logs to be completely, very, or moderately effective.

According to the facilitators, the trigger logs were effective in familiarizing participants with their chewing triggers, and that awareness was helpful for anticipating situations that could tempt them to chew (Table 4). Similarly, facilitators found the self-monitoring logs to be effective in helping participants understand their chewing patterns, linking their feelings with the reasons they chew, and evaluating the extent of their daily use. Facilitators indicated that participants were able to identify physical and social triggers through the use of these logs, and were able to visualize and document behavioral and emotional patterns that lead to chewing.

**Table 4:** Examples of quotations regarding the effectiveness of chewing trigger logs and self-monitoring logs

Trigger Logs		Self-monitoring Logs
**Visual**: Sometimes, the participant is not aware of what triggers them, so having a visual of their triggers logged down helped them prepare. When they put it down in writing, they finally realize their pattern and why they are triggered. **Purposeful**: It helped the participant to be mindful of the triggers as they filled in the log sheets. Now that they have identified their triggers, they are able to work around it. **Revealing**: The logs helped participants to realize the different situations that caused them to chew.		**Pattern**: When participants log their chews instances throughout the day, they can reveal a pattern that they might be unaware of. From there we could find out what their triggers are and plan from there. **Link to chewing/feeling**: This log connected the participants chewing with how they were feeling, the frequency and the association at the time of chewing. Many didn’t realize how often and most especially how they were feeling. **Frequency of Use**: Most of the participants were clueless as to how much they chewed. They were blown away. A few participants mentioned that they were so used to chewing that mixing their chew became a mindless thing– it was done unconsciously.

### Protocol adaptations

#### Evaluations of protocol adaptations

Facilitators were encouraged to adapt the written BENIT protocol as needed. Most facilitators believed it to be at least somewhat acceptable to add material that was not in the written protocol, and to adapt the program based on experience (Table 5). However, all but one facilitator believed it was unacceptable to omit material that was present in the written protocol.

**Table 5:** Level of acceptance on BENIT protocol adaptation

Level of Acceptance	To add information or materials that the written program did not address	To leave out specific material in the written program	To adapt the program as you became more experienced in delivering the program over time
Not Acceptable at all	2	5	2
Somewhat Acceptable	2	0	1
Moderately Acceptable	2	1	2
Very Acceptable	0	0	1
Completely Acceptable	0	0	0

*Note*: Values represent the number of facilitators selecting each session

We also assessed whether facilitators were more likely to adapt certain sessions rather than others. At least one facilitator endorsed each session. Session 2 received the most endorsements (five), followed by session 3 (three endorsements), session 4 (two endorsements), and sessions 1 and 5 (one endorsement each).

Facilitators were also asked about their reason(s) for adapting the BENIT protocol. Two facilitators felt their personal experiences provided them with an informed perspective to adapt the protocol. Four facilitators adapted the protocol because of the participants’ specific attributes (e.g., ethnicity, language). One facilitator adapted the protocol due to perceived time constraints. And three facilitators adapted the protocol based on prior and ongoing experience with the BENIT program (i.e., trial and error).

### Recommended changes

In addition to protocol adaptations, facilitators made several recommendations in the open-ended sections of the survey. These recommendations, listed in Table 6, included a preference for additional time, training, and support. Another recommendation was for clear contingency plans regarding domestic conflicts, natural disasters, and other exigent circumstances.

**Table 6:** Additional recommendations

Additional time	Session times were a constraint to program delivery. All sessions need more time.
Training	More training is needed for Session 3 and session 4.
Support	Facilitators indicated moderate levels of support to address obstacles. Weekly check in with facilitators would help to increase support and reduce burnout.
Contingency plans and process for possible domestic conflicts	Have contingency plans for natural disasters, and clear processes on what to do in the case of domestic conflicts.
Interventions and Facilitators	Increase strategic preparation and training for the randomized trial. Hire certified cessation facilitators (nicotine, brief tobacco intervention) to deliver the betel nut cessation training and facilitation. Consider former chewers as facilitators.
Cultural adjustment	Adjust program to be culturally sensitive regarding the social aspects of chewing

## Discussion

The BENIT program was generally perceived to be effective and well received, and many of the program’s components worked as intended. Trigger and self-monitoring logs, for example, provided visual reminders that helped participants understand their chewing patterns and thereby foster behavior changes and coping strategies to deal with chewing urges in ways that were meaningful to them. However, other program components, such as session 3 and 4, did not proceed as smoothly as anticipated, and the facilitators provided suggestions for improvement. These suggestions included hiring experienced facilitators, and adding more time and training to comprehensively address the sessions’ vital topics, which included withdrawal symptoms, coping strategies, and additional strategies to deal with chewing urges.

Most of the facilitators felt moderately prepared to deliver the BENIT program after training. However, following the program’s completion, some facilitators indicated sessions for which they could have been better prepared. For instance, knowing how to deal with cultural and language barriers, and how to better manage time, particularly for sessions 1 and 2, would have been helpful.

Regarding the written BENIT protocol, facilitators mostly adhered to the program as it was intended to be delivered, though made moderate adjustments as needed due to cultural considerations and time management issues during the sessions. The facilitators did not feel that any part of the written program should be omitted, but believed that changes could be made when time constraints intervened or when cultural considerations necessitated adjustments. Facilitators also believed that it was acceptable to make changes to the protocol as they became more experienced and grew more confident in administering the program.

Additional support is needed to help the facilitators overcome obstacles such as burnout and stress. The feedback provided by the facilitators is understandable. The BENIT was administered with a limited budget and within a limited timeframe. Though the facilitators received training beforehand, the role as cessation counselors was still new to them. In addition, most facilitators also took on the additional role of study coordinator. Future versions of BENIT should carefully consider the importance of facilitator training and experience, and budget accordingly.

A lot of preliminary work went into developing the BENIT; however, it is a novel program and, thus, remains a work in progress due to the current lack of evidence-based ANBQ cessation programs. It was vial to learn as much as we could from the people who administered BENIT. In this way, we learned not just about the essential outcomes of BENIT (i.e., cessation rates), but also how the program was experienced by the people most directly involved with its administration.

## Conclusion

Administering the BENIT program was a demanding and stressful undertaking, but at the same time a fulfilling and worthwhile experience. While some program components worked as intended, others could have proceeded more smoothly if given additional time and training. The current findings will be used to improve future versions of BENIT and to encourage other interested investigators to further develop ANBQ cessation interventions.

## Data Availability

The data generated and/or analyzed during the current study are not publicly available due to privacy and ethical considerations, but are available from the corresponding author on reasonable request.
